# Clinical Effects of Immersive Multimodal BCI-VR Training after Bilateral Neuromodulation with rTMS on Upper Limb Motor Recovery after Stroke. A Study Protocol for a Randomized Controlled Trial

**DOI:** 10.3390/medicina57080736

**Published:** 2021-07-21

**Authors:** Francisco José Sánchez-Cuesta, Aida Arroyo-Ferrer, Yeray González-Zamorano, Athanasios Vourvopoulos, Sergi Bermúdez i Badia, Patricia Figuereido, José Ignacio Serrano, Juan Pablo Romero

**Affiliations:** 1 Facultad de Ciencias Experimentales, Universidad Francisco de Vitoria, 28223 Pozuelo de Alarcón, Spain; fjose.sanchez@ufv.es (F.J.S.-C.); aida.arroyo@ufv.es (A.A.-F.); 2 Escuela Internacional de Doctorado, Department of Physical Therapy, Occupational Therapy, Rehabilitation and Physical Medicine, Universidad Rey Juan Carlos, 28933 Alcorcón, Spain; y.gonzalezz@alumnos.urjc.es; 3 Institute for Systems and Robotics-Lisboa, Department of Bioengineering, Instituto Superior Técnico, Universidade de Lisboa, 1049-001 Lisbon, Portugal; athanasios.vourvopoulos@tecnico.ulisboa.pt (A.V.); patricia.figueiredo@tecnico.ulisboa.pt (P.F.); 4 Faculdade de Ciências Exatas e da Engenharia, Madeira Interactive Technologies Institute, NOVA LINCS, Universidade da Madeira, 9020-105 Funchal, Portugal; sergi.bermudez@uma.pt; 5 Neural and Cognitive Engineering Group (gNeC), Centre for Automation and Robotics (CAR), Spanish National Research Council (CSIC-UPM), 28500 Arganda del Rey, Spain; jignacio.serrano@csic.es; 6 Brain Damage Unit, Beata María Ana Hospital, 28007 Madrid, Spain

**Keywords:** stroke, repetitive transcranial magnetic stimulation, BCI-VR training, motor skills, upper limb

## Abstract

*Background and Objectives:* The motor sequelae after a stroke are frequently persistent and cause a high degree of disability. Cortical ischemic or hemorrhagic strokes affecting the cortico-spinal pathways are known to cause a reduction of cortical excitability in the lesioned area not only for the local connectivity impairment but also due to a contralateral hemisphere inhibitory action. Non-invasive brain stimulation using high frequency repetitive magnetic transcranial stimulation (rTMS) over the lesioned hemisphere and contralateral cortical inhibition using low-frequency rTMS have been shown to increase the excitability of the lesioned hemisphere. Mental representation techniques, neurofeedback, and virtual reality have also been shown to increase cortical excitability and complement conventional rehabilitation. *Materials and Methods:* We aim to carry out a single-blind, randomized, controlled trial aiming to study the efficacy of immersive multimodal Brain–Computer Interfacing-Virtual Reality (BCI-VR) training after bilateral neuromodulation with rTMS on upper limb motor recovery after subacute stroke (>3 months) compared to neuromodulation combined with conventional motor imagery tasks. This study will include 42 subjects in a randomized controlled trial design. The main expected outcomes are changes in the Motricity Index of the Arm (MI), dynamometry of the upper limb, score according to Fugl-Meyer for upper limb (FMA-UE), and changes in the Stroke Impact Scale (SIS). The evaluation will be carried out before the intervention, after each intervention and 15 days after the last session. *Conclusions:* This trial will show the additive value of VR immersive motor imagery as an adjuvant therapy combined with a known effective neuromodulation approach opening new perspectives for clinical rehabilitation protocols.

## 1. Introduction

Stroke is a leading cause of long-term disability; it reduces mobility in more than half of stroke survivors age 65 and over [1].

Despite the lack of objective prognostic factors regarding the patient’s functionality after a stroke, we know that age, the level of initial disability, and the location and size of the lesion are elements that affect the evolution of post-stroke rehabilitation [2].

After a stroke, the recovery of lost functions in the brain is achieved thanks to reorganizing networks in a process known as plasticity. Some damaged brain tissues may recover, or undamaged areas may take over some functions.

One of the most relevant aspects of the rehabilitation prognosis is the time of evolution. After a stroke, improvement is noticeably reduced over the second month, finding stabilization around the sixth month. One of the reasons that explain this fact is the reduction of neuroplasticity [3]. There are indicative studies that reflect that, six months after a stroke, more than 60% of subjects will have a non-functional hand for Basic Activities of Daily Living (BADL), and 20–25% will not be able to walk without assistance [4]. These impairments determine the important global burden that stroke represents [5]. It is relevant to emphasize that the degree of disability after the rehabilitation process will be determined by the combination of existing motor, sensory, and neuropsychological deficiencies [6].

In the last years, several non-invasive neuromodulation techniques have been shown efficient to enhance plasticity and stroke recovery. Among these interventions, we can find exogenous neuromodulation, meaning that the neuromodulator stimulus comes from an external source, as is the case with rTMS (repetitive transcranial magnetic stimulation), which has the capacity to change the cortical excitability depending on the frequency of the magnetic pulses. Low frequencies (≤1 Hz) reduce local neural activity, and high frequencies (≥5 Hz) increase cortical excitability [7]. This technique has been successfully used bilaterally, stimulating the injured hemisphere and inhibiting the healthy one, to treat the interhemispheric inhibition phenomenon in stroke patients as it influences stroke recovery [8]. Although up to date there are some consensus recommendations about dosing and parameters of rTMS in some clinical applications such as pain [9] and depression [10], there is no clear consensus about the recommended parameters in stroke due to the heterogeneity of the currently published studies [11].

On the other hand, there are endogenous neuromodulation techniques that depend on the capacity of the subject to modulate its own brain activity. This can be achieved using neurofeedback (NFB), which consists of recording information of brain activity using electroencephalography (EEG) or functional magnetic resonance (fMRI) and displaying it to the subject in such a way that he can receive real-time information of his own brain function. Virtual reality allows a new dimension on the neurofeedback immersion and is likely to increase its efficacy [12]. Stroke patients have been trained to reinforce certain EEG rhythms related to motor performance using the NFB technique showing favourable effects on rehabilitation outcomes [13].

Some other techniques aiming to increase brain plasticity use the practice of imagination of movement of the affected hemibody [14]. This is known as motor imagery [15] and can also be enhanced through the use of brain–computer interfaces [16]. All the neuromodulation techniques are used to complement but not as a replacement of conventional rehabilitation [17,18].

On the one hand, exogenous neuromodulation effects are produced mainly by changes directly induced in cortical excitability [19], and on the other hand, endogenous neuromodulation is believed to have more widespread subcortical effects [20,21]. Currently, the exact mechanisms underlying motor recovery using neuromodulation techniques are still being investigated. Simultaneous activation of inputs and outputs to cortical motor areas is believed to trigger Hebbian plasticity, which strengthens cortical-subcortical connectivity. Improvements in this cortical-subcortical connectivity have been linked to better motor recovery after stroke [22,23].

One of the probable causes of the short-term effects of these techniques is the ceiling effect of changes in cortical excitability that can be achieved non-invasively, but despite the good results achieved with the use of non-invasive neuromodulation techniques individually, there is a shortage of validated neurorehabilitation protocols that integrate different approaches that have been proven to be effective individually.

It has been suggested rTMS may be a good conditioning method whose effect would be to increase brain plasticity that would improve the results of subsequent rehabilitation [24]. There are already several studies that compare rTMS alone with its combination with motor rehabilitation showing good results when rTMS is used as a primer that may help to reach a greater cortical activation and boosting plasticity [8,25,26,27]. Motor imagery and action observation have been shown to effectively activate the same brain areas as an actual movement [28]; up to date, it has not been combined with rTMS.

There is increasing evidence of Brain–Computer Interfacing (BCI) efficacy as rehabilitation technology in patients with severe motor impairments [29]. This is the case with NeuRow [30]. It is an immersive multimodal Brain–Computer Interfacing (BCI) training paradigm that combines motor imagery and embodied neurofeedback using virtual reality. NeuRow has been designed to be used by the chronic stroke patient population [31], and its efficacy has been shown in a pilot study. It shows clear improvements and recovery regarding motor function in terms of clinical scales (FMA, MAS, SIS), self-reported scales, electrophysiological data, and brain imaging data (fMRI). There is evidence that BCI-based rehabilitation promotes lasting improvements in motor function in chronic stroke patients with hemiparesis [32].

Both approaches, the NeuRow training paradigm (NeuroRehabLab, Funchal, Portugal) and bilateral rTMS protocols, are likely to complement their effects, achieving a stronger neuroplasticity enhancement in stroke patients. Both have been used separately for the treatment of motor sequelae in the upper limbs after stroke [8,31,33]. The effects of these combined techniques are not likely to be based only on the increase of cortical excitability but also on subcortical mechanisms.

The efficacy of rTMS and motor imagery has been previously demonstrated, but their combination has not been tested yet. The main objective of this study is to carry out a double-blind, randomized, controlled trial aiming to study the clinical effect of immersive multimodal BCI-VR training using NeuRow system (NeuroRehabLab, Funchal, Portugal) after bilateral neuromodulation with rTMS compared to bilateral rTMS plus conventional rehabilitation in upper limb motor sequelae after subacute stroke (>3 months). We will look for changes in: 1. isometric strength in the upper limb; 2. functional motor scales of the upper limb; 3. hand dexterity; and 4. cortical excitability changes. Our main hypothesis is that multimodal BCI-VR training will be superior to the use of conventional motor imagery as adjuvant therapy to bilateral rTMS.

This protocol combines techniques that have proven to be cost-effective [34]. If it is shown that the clinical improvement with this combination is significant, it will open a new line of combined neuromodulation approaches to reach an effective method for the upper limb motor neurorehabilitation after a stroke.

## 2. Materials and Methods

The SPIRIT 2013 Checklist has been used to assure the quality of the protocol [35]. This protocol has been registered in trials.gov with the number NCT04815486.

### 2.1. Study Design and Participants

Participants will be recruited in the Brain Injury Unit or Rehabilitation Unit of the Beata María Ana Hospital; patients referred from other centres and self-referred patients will also be considered. The subjects included will be assessed by a neurologist (JPR) and a physiotherapist (FJS). All patients will have had a hemispheric ischemic or hemorrhagic stroke (>3 months after stroke) diagnosed in at least one brain-imaging test and presenting motor sequelae in the upper limb. We have ruled out the first three months since the phenomenon is known as “spontaneous recovery” [36] takes place in time, and therefore, it would be difficult to discern the cause of the clinical improvement that we expect from patients. The patients included in the study will have an alteration of mobility and/or functionality in the upper limb related to the stroke and with a score equal to or greater than 25 according to the Fugl-Meyer Assessment for upper extremity (FMA-UE) [37]. The rest of the specific inclusion and exclusion criteria are shown in Table 1.

The study design corresponds to a randomized, single-blind, controlled clinical trial in which patients are randomly assigned to two groups: 1. Conventional rehabilitation + bilateral rTMS + Immersive multimodal BCI-VR training system NeuRow (NeuroRehabLab, Funchal, Portugal), and 2. Conventional rehabilitation + bilateral rTMS.

Regarding adherence strategies, sessions missed up to the established limit will be restored the following week. Flexible therapy schedules will also be offered, and patients’ families will be contacted directly by phone to confirm evaluation dates, thus reinforcing treatment adherence [38].

To calculate the sample size, the GRANMO calculator was used *(Sample size and power calculator (v. 7.12.), Institut Municipal d’Investigació Mèdica, Barcelona, Spain)*. Accepting an alpha risk of 0.005 and a beta risk of 0.2 in a one-sided contrast, 23 subjects are required to detect a difference equal to 4.9 points, the minimal clinically important difference of the Fugl-Meyer assessment scores for wrist/hand [39]. Considering a 15% loss, it will be necessary to reach a sample of 21 patients in each group.

Randomization and blinding will be made using the application Research Randomizer (Social Psychology Network, Middletown, CT, USA) [40] to form the groups. A code consisting of two digits (1 and 2) will be used. The application offers a random list of 30 numbers with digits 1 and 2. This sequence will be carried out remotely by a blind researcher who is not involved in other investigation procedures. After the randomization process, another blind staff member will assign patients among groups. The allocation concealment will be made with closed, sealed, and sequentially numbered envelopes. The evaluators will receive the patients in a different room, ignoring which group they belong to.

To assess the adverse effects, participants will be asked at the end of each session if they experienced effects such as tingling, burning, headache, and drowsiness, among others and the intensity of this sensation. Guidelines for safety on rTMS protocols will be followed [41]. However, no severe adverse effects are expected. Any adverse effect will be notified to a licensed medical doctor. Management of possible adverse effects will be individualized and according to the severity.

Regarding data processing security, data will be recorded separately and will be anonymized and guarded following current European data protection laws. All data will be recorded and verified twice in a database specifically designed for the studies.

### 2.2. Intervention Protocol

The two intervention protocols have been extracted from previous publications of successful application on upper limb rehabilitation. On the one hand, the investigation by Takeuchi et al. showed significant improvements in those subjects who received bilateral rTMS stimulation (based on the principle of interhemispheric inhibition) [8]. On the other hand, Vourvopoulos et al. published a pilot study using NeuRow, showing a high performance [33].

The intervention will consist of the two therapies administered sequentially on the same day rTMS application before the immersive multimodal BCI-VR training, or just rTMS application, in the control group. The sequence of administration has been decided based on preliminary results of the NeuroMOD project (unpublished) from our group, revealing that patients receiving rTMS prior to NFB had a better performance.

#### 2.2.1. rTMS Stimulation

Firstly, single-pulse TMS will be performed to acquire a resting motor threshold (rMT). Magstim Rapid2 (Magstim Company, Whitland, Wales, UK) device with an air-cooled 70 mm figure-of-eight magnetic stimulator coil will be used. For all assessments, the magnetic stimulator coil will be placed on M1 in the assessed hemisphere, and a surface electromyogram from the contralateral first dorsal interosseous muscle will be recorded using surface EMG using CED Signal Software (CED, Cambridge, UK). EMG recordings will be recorded and stored in a computer for offline analysis. Stimulation output intensity used to reach the resting motor threshold (RMT) will be used to calculate the parameters for the stimulation sessions.

rTMS parameters will be as described by Takeuchi et al. [8]: 90% of the RMT at 10 Hz, 1000 pulses, 5 s intertrain interval on M1 of the lesioned hemisphere and after 5 min rest period, the contralateral M1 area will be stimulated with 90% of the RMT at 1 Hz, 1000 pulses, with a 50 s intertrain interval. This will be performed in 10 consecutive daily sessions (Monday to Friday).

#### 2.2.2. Immersive Multimodal BCI-VR Training

NeuRow is a gamified BCI training paradigm in VR that allows patients to perform similar motor actions as they would do in real life. NeuRow is rendered through a head-mounted VR headset with 90° horizontal field-of-view and haptic feedback delivered through 2 controllers in both hands.

The paretic limb should be positioned in a resting position on the table. Before setting up the EEG cap plus VR headset in each session, a previous training will be carried out with the following instructions:

1. Ask the patient to perform the rowing movement with both upper limbs with external facilitation of the paretic side.
2. Ask the patient to imagine the movement with eyes closed, focusing on his internal perspective and on the sensation of rotation. Imagine the hand closed in a fist and feel the arm weight and contraction of arm muscles.
3. Imagine the movement slowly and increase their speed.
4. The best strategies will be identified for each participant. The patient reports in detail what he felt/tried to visualize; the researcher will give feedback and will also give a description of the sensation of the movement to the participant during the motor imagination, describing the sequence of movements required for rowing (elbow stretched, closed hand grasping the paddle, etc.).
5. The patient will be asked if he succeeds in imagining the tasks.

It is very important that the movement is natural and biomechanically correct. This training will be carried out daily and prior to the application of the NeuRow system.

**Figure 1:**
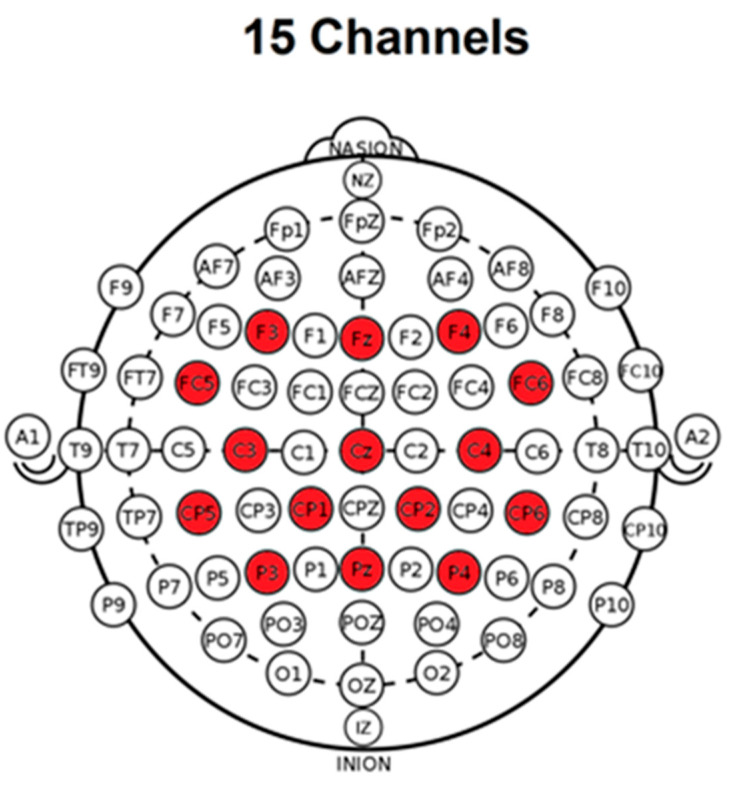
The spatial distribution of the electrodes. In red, the active electrodes.

Before the actual VR training, the acquisition of the EEG will be carried out. An experienced technician will acquire EEG through a BCIs system with 64 active electrodes equipped with a low-noise biosignal amplifier and a 24 bit A/D converter at 256 Hz (BrainVision actiCHamp biosignal amplifier, Brain Products GmbH, Gilching, Germany). The spatial distribution of the electrodes will cover primarily the motor and somatosensory areas of the brain. Specifically, the Frontal (F3, Fz, F4), Frontal-Central (FC5, FC6), Central (C3, Cz, C4), Central-Parietal (CP5, CP1, CP2, CP6), and Parietal (P3, Pz, P4) in a small Laplacian configuration for spatial filtering (Figure 1). EEG data acquisition and processing will be performed through the OpenVibe platform [42], which will transmit the data via the Lab Streaming Layer (LSL) protocol to control the virtual environment.

Secondly, the BCIs training protocol designed and adapted based on the Graz-BCI paradigm will be used [43]. The first step will be the acquisition of the raw EEG data to extract features to train a classifier to distinguish Right and Left imagined hand movements. Thus, the patient will have to perform mental imagery of the corresponding hand according to the presented stimuli on the screen. The training session will be configured to acquire data in 24 blocks per class (Right-or Left-hand imagery) in a randomized order. Afterwards, the data will be filtered both spatially and temporarily between the Alpha and Beta bands (8–30 Hz) for creating the feature vector.

During the training session, patients will wear a VR headset and see a boat and two high fidelity virtual arms gripping two oars in the first-person view; they will have to imagine the movement of each corresponding hand to rotate each oar and progress, observing the movement imagined on screen [31]. The game interface includes timekeeping and scoring. The goal of the task is to perform as many correct Motor Imagery sequences as possible in a fixed amount of time. To improve adherence, points will be awarded depending on the performance in each session [33,34]. The Motor Imaging training mode will be used. Training sessions will be performed in 12 non-consecutive sessions (Monday, Wednesday and Friday, during four weeks) lasting 30 min each, divided into 3 series of 7 min including initial training and break time to prevent fatigue [31].

### 2.3. Outcomes Measurement

**Figure 2:**
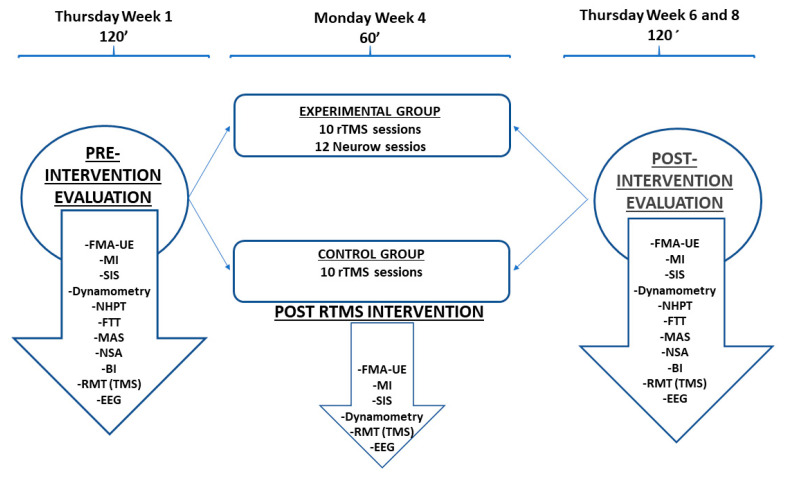
Schematic representation of intervention protocol. It includes the days of the week and duration of each block of outcomes measurement in pre- and post-evaluation (FMA-UE—Fugl-Meyer Assessment for upper extremity; MI—Motricity Index; SIS—Stroke Impact Scale; RMT—Resting Motor Threshold; NHPT—Nine Hole Peg Test; FTT—Finger Tapping Task; MAS—Modified Asworth Scale; NSA—Nottingham Sensory Assessment; BI—Barthel Index; EEG—Electroencephalogram. Thursday/90–120 min; Monday/60 min; Control Intervention/Monday to Friday/10 rTMS sessions and Experimental Intervention: Monday to Friday/10 rTMS sessions and Monday, Wednesday, and Friday/12 NeuRow sessions).

Three evaluations will be carried out for each patient. A pre-intervention initial evaluation, a second evaluation the week following the end of the rTMS intervention, and a final evaluation after two weeks, when the NeuRow training finish. The first and third evaluation sessions will have an average duration of 120 min, and the second evaluation will have a duration of 60 min (Figure 2).

#### 2.3.1. Main Outcomes

Due to the bilateral stimulation protocol used, all outcomes will be assessed on both sides.

Motricity Index of the Arm (MI): The upper limb section of the MI assesses muscle strength in 3 muscle groups, including grip, elbow flexion, and shoulder separation. Each movement is scored discreetly (0 if there is no movement, 9 if the movement is palpable, 14 if the movement is visible, 19 if the movement is against gravity, 25 if the movement is against resistance, and 33 if the movement is normal), obtaining a total score for the upper limb that ranges from 0 (severely affected) to 100 (normal). This assessment methodology has been widely used in rehabilitation progress evaluation [44,45] and counts with a normalized and weighted scoring system.

Dynamometry: A handheld analogic dynamometer (Jamar^®^ Plus+ Hand Dynamometer, 0–90 kg) (Performance Health Supply, Nottinghamshire, UK) will be used to assess isometric grip strength. Patients will be positioned in a straight back chair with both feet on the floor and the forearm resting on a stable surface. Patients will perform a maximal isometric grip contraction until they reach maximal force output. Three measures will be taken with 1 min rest between tests, and the mean value will be recorded. This provides an objective evaluation of handgrip strength that will allow pre- and post-protocol comparison.

Fugl-Meyer Assessment for upper extremity (FMA-UE): The FMA-UE is an observational rating scale that assesses sensorimotor impairments in post-stroke patients. It also includes four subscales: A. Upper Extremity (0–36), B. Wrist (0–10), C. Hand (0–14), and D. Coordination/Speed (0–6), composing a total maximum score of 66 points. This scale is considered adequate to detect changes in motor recovery in patients who have suffered a stroke because of its wide evaluation items [46]. The minimum clinically important difference (MCID) for the upper limbs has been determined and ranges from 4.25 to 7.25 points, depending on the evaluated region of the upper limb [47].

Stroke Impact Scale (SIS): The SIS is a stroke-specific quality of life instrument to assess the quality-of-life impairment after stroke only considering the physical domain. It presents 4 subscales, but only the hand function domain will be evaluated. The SIS is a brief and easy instrument that considers specifically motor impairment impact over daily living activities [37].

#### 2.3.2. Secondary Outcomes

Computerized Finger Tapping Task (FTT): The FTT measures motor function and is very sensitive to the slowing down of responses. In this task, following the Strauss application norms, the participants will be instructed to sit comfortably in front of a computer and press the spacebar on the keyboard as fast as possible and repeatedly with the index finger. Five 10 s attempts will be performed with each hand. The average time between two consecutive taps in the five trials will be the dependent variable.

Nine Hole Peg Test (NHPT): The NHPT evaluate the impairment in upper limb dexterity [48]. Patients must pick up as quickly as possible nine pegs from a container one by one unimanually and transfer them into a target pegboard with nine holes until filled. Then, they must return them unimanually to the container. The outcome variable will be the time spent to complete the whole task [49]. The NHPT is considered reliable [50], valid, and sensitive to change [51] among stroke patients.

Modified Ashworth Scale (MAS): The MAS is one of the most used tools for the assessment of spasticity [52]. Patients will be in the supine position with their arms by their side and with their head in a neutral position [53]. The MAS is markedly responsive in detecting the changes in muscle tone in patients with stroke [54]. This scale will be used because secondary changes in spasticity are possible if motor changes are produced.

Nottingham Sensory Assessment (NSA): Somatosensory impairment of the upper limb occurs in approximately 50% of adults after stroke, associated with loss of hand motor function, activity, and participation. The measurement of sensory impairment in the upper limb is a component of rehabilitation that contributes to the selection of sensorimotor techniques that optimize recovery and provide a prognostic estimate of the function of the affected upper limb [55]. There are studies documenting changes produced in the sensation of the upper limb after the application of neurofeedback [56] and even after the intervention with motor imagery [57]. Since the protocol presents an intervention with the application of these techniques, it is possible that there will be changes related to the sensitivity after the use of the platform, NeuRow system (NeuroRehabLab, Funchal, Portugal) [31].

Barthel Index (BI): Accurately assessing the ADLs of stroke patients greatly helps in evaluating the efficacy of stroke treatments [58]. The Barthel Index was originally established to assess ADL in stroke patients and has been used extensively for this purpose [59].

Neurophysiological measurements of cortical plasticity changes:

TMS Resting Motor Threshold (RMT) in the first dorsal interosseous muscle or the abductor pollicis brevis muscle will be recorded to determine the cortical excitability changes and correlate them with the clinical outcomes.

EEG: Different measures of quantitative EEG will be collected. They have been shown to be very useful in evaluating stroke patients’ recovery [60].

### 2.4. Data Analysis

Parametric tests will be used for the analysis of the results if compliance with the assumptions (normality and equality of variance) and the sample size allow it. These analyses include a Student’s *t*-test for independent or paired samples, ANOVA of one or two factors and repeated measures, and Pearson correlations.

Due to the data nature, analyses will be completed with nonparametric tests such as χ2 and Wilcoxon. The residual effect, period effect, and sequence effect checks will be made.

In all analyses, a confidence level of 0.95 will be adopted. The data analysis will be carried out with the help of the statistical program SPSS 25.0 (SPSS Inc., Chicago, IL, USA).

### 2.5. Dissemination Plans

All results will be published in specialized scientific journals. Results will be made public through the social media of our institution.

## 3. Discussion

Non-invasive brain stimulation (NIBS) by repetitively activating circumscribed brain regions with magnetic stimulation has a promising future as an augmentative therapeutic approach to traditional physical therapy after stroke [61]. rTMS protocols based on interhemispheric inhibition compensation have been reported to compensate for this phenomenon and consolidate neuroplastic changes [62].

On the other hand, the results from the studies by Vourvopoulos et al. [31] and Ramos-Murguialday et al. [32], using multimodal immersive BCI-VR training, have also shown to improve the carry-over effect of the rehabilitation process evidenced in clinical scales, self-reported scales, electrophysiological data, and brain imaging data.

Several previous studies combining two different non-invasive neuromodulation approaches have been successfully tested in stroke patients’ rehabilitation [63,64,65]. This is the first time that multimodal immersive BCI-VR training is evaluated as an enhancer of the proven efficacy or rTMS bilateral protocols focused on interhemispheric inhibition compensation.

It seems that in the rehabilitation of the hand after a stroke, the cortico-subcortical connectivity mechanisms are relevant [23]. Exogenous source neuromodulation (rTMS) constitutes a cortical excitability input that activates the cortex and cortico-spinal pathway in a top-down mechanism. On the other hand, endogenous neuromodulation (motor imagery) may be similar to bottom-up rehabilitation mechanisms elicited by physical motor activation activating cortical and subcortical mechanisms [66]. In this way, we think that by combining both therapies, we will further enhance the cortico-subcortical connectivity, and therefore, the clinical effects regarding the motor recovery of the affected limb will be greater.

The combination of these techniques with the extensive, objective evaluation of upper limb outcome will generate a new hypothesis about how the combination of different neuromodulation approaches affect homeostatic plasticity and, thus, motor recovery.

The validation of this protocol will determine the clinical utility of the combination of two non-invasive neuromodulation approaches to enhance the effect of conventional rehabilitation on stroke. The outcomes of this study will contribute to identifying if multimodal immersive BCI-VR training enhances the known effects of rTMS over interhemispheric inhibition. Neurophysiological and clinical prognostic factors of response to this protocol will be determined.

## 4. Conclusions

This trial will show the additive value of VR immersive motor imagery as an adjuvant therapy combined with a known effective neuromodulation approach opening new perspectives for clinical rehabilitation protocols. This therapy could potentially reduce the time required for hand rehabilitation or improve functional outcomes reducing long-term disability.

## Figures and Tables

**Table 1:** Inclusion and exclusion criteria.

INCLUSION CRITERIA	EXCLUSION CRITERIA
Older than 18 years old.	History of seizure or brain aneurysm
Ischemic or hemorrhagic cerebrovascular injury diagnosed by a neurologist and who have at least one brain-imaging test	Pacemakers, medication pumps, metal implants in the head (except dental implants)
Onset of hemispheric ischemic or hemorrhagic stroke >3 months	Clinical instability
Kinesthetic and Visual Imagery Questionnaire (KVIQ) >55.	Muscle tone in the wrist and hand with a modified Ashworth scale (MAS) score equal to or higher than 3 in the wrist
Stability in antispastic medication for more than 5 days	Other pre-existing neurological diseases or previous cerebrovascular accidents with sequelae
Able to read and write	Aphasia
Sufficient cognitive ability to understand and perform tasks: Token Test >11	Previous TMS after stroke Hemispatial neglect (Bells Test >6 omissions on one side) Visual problems
	Flaccid paralysis Brunnstrom’s stage = 1

## Data Availability

The data presented in this study will be available on request from the corresponding author.
